# Transcorneal Permeation in a Corneal Device of Non-Steroidal Anti-Inflammatory Drugs in Drug Delivery Systems

**DOI:** 10.2174/1874104500802010066

**Published:** 2008-05-22

**Authors:** R Valls, E Vega, M.L Garcia, M.A Egea, J.O Valls

**Affiliations:** Department of Physical Chemistry, Faculty of Pharmacy, University of Barcelona, Spain; Institute of Nanoscience and Nanotechnology, University of Barcelona, Spain

## Abstract

This work is focused on the *ex vivo *study of corneal permeation of two anti-inflammatory drugs: diclofenac, and flurbiprofen (as a model of hydrophilic and lipophilic drug, respectively) loaded to cyclodextrins or polymeric nanoparticles in order to determine differences in their corneal permeation against free drug or commercial eye drops. These studies were carried out in a corneal device designed and developed in our laboratory. In this work the habitual conditions for the permeation studies were modified to reproduce the behaviour when eye drops were administered. For this reason a new tetracompartmental pharmacokinetic model was developed. The complex formation of diclofenac with cyclodextrins and the flurbiprofen loaded to polymeric nanoparticles has been shown as effective procedures to remarkably increase the bioavailability of the anti-inflammatory drugs. The efficiency of polymeric nanoparticles of Poly (D-L lactic-coglycolyc) acid and poly-ε-caprolacton as intraocular targeting of NSAIDs has also been proved, being the latter polymer more effective to increase the flurbiprofen corneal permeation. The apparent corneal permeability coefficient of samples has been calculated getting a low permeation values for free drugs.

## INTRODUCTION

Nonsteroidal anti-inflammatory drugs (NSAIDs), like diclofenac and flurbiprofen, have been found to be viable alternatives to steroids in treating ocular inflammation. This therapy requires, due to low permeation rates and a rapid pre-corneal loss, a frequent application or highly concentrated eye drop formulations. The drugs must penetrate across the cornea, to reach therapeutic targets within the globe. In general, no more than five percent of drug, present in the precorneal area, crosses the cornea and arrives to intraocular tissues. The rest of the administered drug is dragged by the tear to the nasal conduit where it is eliminated later by digestive tract.

The present research was focused on the *ex vivo *study of corneal permeation of two anti-inflammatory drugs (NSAIDs), frequently used in ocular pharmacology: diclofenac and flurbiprofen. The corneal permeation of these free drugs was compared to the one of the diclofenac forming complexes with cyclodextrins or the flurbiprofen loaded to polymeric nanoparticles.

## MATERIALS AND METHODS

These studies were carried out in a corneal device designed and developed in our laboratory for this research [1], based in the others previously described [2-4].

The Fig. (**1**) shows the chamber used to measure corneal permeation in detail.

The principle of the apparatus is that the cornea of the rabbit is placed in a methacrylate chamber clamped between two pieces so that the epithelial surface faces one compartment (anterior or tear compartment) and the endothelial surface the other (posterior or aqueous humor compartment).

The Fig. (**2**) shows the entire setup of the system. The fluid contained in the anterior compartment can be exchanged continuously by means of a pump. The hydrostatic pressure in endothelial compartment can be chosen arbitrarily and independently. The experimental temperature can be controlled continuously.

The transcorneal electrical potential can be measured and registered also continuously to check the vitality of the rabbit cornea [5, 6].

Albino New Zealand rabbits weighing 1.8 to 2.2 kg, were used. After rabbits were sacrificed, the corneas were removed and immediately mounted and clamped in the chamber.

The basic solutions were prepared from reagent grade chemicals as in the report of O’Brien and Edelhauser [7].

The artificial tears solution (ATS), for the epithelial side of the cornea, was a Bicarbonate buffered Ringer's solution prepared previously and maintained at 4º C. Previously to perform experiment the above solution was warmed and was added 0.9 g L^-1^ of glucose with magnetic stirring.

To prepare the artificial aqueous humor (AAH) for the endothelial side of the cornea takes the obtained ATS and add Glutathione 0.13 g L^-1^ and Adenosine 0.09 g L^-1^ at 37º C with stirring just before use.

The extraction of small volumes of the endothelial solution without loss of pressure was carried out by means of a syringe with a needle that crosses the cork of the endothelial compartment. Samples of 50 μL of the endothelial compartment were taken at different times (between 5 minutes and 2 hours) and then were analysed by Ultraviolet Absorption Spectroscopy.

Apparent permeability coefficients *(Papp) *were measured by previously described methods [8], according to this equation:

$P_{\mathrm{app}}=\frac{\delta \text{Q}}{\delta \text{t}60\text{A}{\text{C}}_{0}}$

where δQ / δt is the variation of drug amount, corresponding to the slope of the linear portion of the graphic (normally the first hour), 60 the conversion from minutes to seconds, A is the corneal surface (in this study 0,55 cm^2^) and C_0_ the initial drug concentration in the epithelial side.

## CORNEAL PERMEATION WITHOUT WASHING PROCEDURE: TRI-COMPARTMENTAL MODEL

Most corneal penetration studies have been performed maintaining constant drug concentration in the artificial tear solution of the external or epithelial compartment. In this way the drug concentration at the internal or endothelial corneal side, increases regularly following an exponential slope like as reflected in Fig. (**4**).

**Fig. (3):**
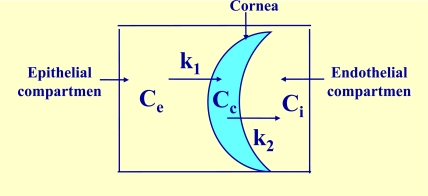
Tricompartmental model. Ce: constant external or epithelial concentration, Cc: variable internal corneal concentration, Ci: variable internal or endothelial concentration, K_1_ and K_2_: transference constants from epithelial compartment to cornea and from cornea to endothelial compartment, respectively.

When there exists a constant flux of drug a simplified, but very precise, tricompartmental model can been used. This model has been developed based on the one proposed by Maurice [9]. It works with the following elements (Fig. **3**).

A constant external concentration Ce, a variable corneal concentration Cc, a variable internal concentration Ci, a transfer rate constant from the exterior to the cornea k_l_, and a transfer rate constant from the cornea to aqueous humor k_2_.

In this work Ce and Ci are known. k_l_ and k_2_ are unknown, while Cc is not needed for the ca1culation. The drug flow is considered to be passive and against the concentration gradient. The statement of apparent corneal permeability can be laid out with the following two kinetic equations:

$\frac{dC_{c}}{\mathrm{dt}}=k_{1}\left(C_{e}-C_{c}\right)-k_{2}\left(C_{c}-C_{i}\right)$

$\frac{dC_{i}}{\mathrm{dt}}=k_{2}\left(C_{c}-C_{i}\right)$

The system is reduced to the following equation:

$C_{i}+\left(k_{1}+2\cdot k_{2}\right)\cdot C_{i}+k_{1}\cdot k_{2}\cdot C_{i}=k_{1}\cdot k_{2}{\cdot C}_{e}$

This equation has the solution:

$y=A\cdot e^{m_{1}.t}+B\cdot e^{m_{2}.t}+C$

where:

$m_{1}=\frac{-k_{1}-2k_{2}+\sqrt{{k_{1}}^{2}+{4k_{2}}^{2}}}{2}$

$m_{2}=\frac{-k_{1}-2k_{2}-\sqrt{{k_{1}}^{2}+{4k_{2}}^{2}}}{2}$

Using the Pythagoras theorem, it can be seen that m_1_ is always negative, as m_2_ where all terms are negative, so we can use n_1_ and n_2_ as m_1_ and m_2_. Taking also into account that C_l_ (0) = 0 and C_i _(t → ∞) = C_e_ the model is simplified obtaining the equation in its final form:

$C\left(i\right)=A\cdot \left[e^{-n_{1}\cdot t}-e^{-n_{2}\cdot t}\right]+{\text{C}}_{\text{e}}\cdot \left[1-e^{-n_{2}\cdot t}\right]$

Were “t” is the variable time and n_1_ and n_2_ two pharmacokinetic parameters related to k_1_ and k_2_. A does not have an immediate pharmacokinetic significance. It depends on the experimental conditions and on the product itself.

**Fig. (4):**
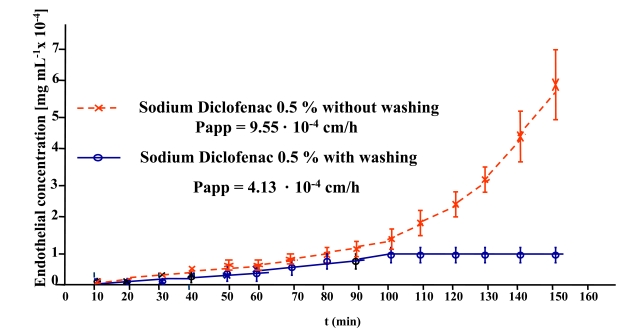
Cornea permeation profile of Sodium Diclofenac 0.5 % solution without washing or with washing.

The apparent permeability for Sodium Diclofenac calculated by the slope of the graphic at sixty minutes was 9.55 · 10^-4^ cm/h (Fig. **4**).

## CORNEAL PERMEATION WITH WASHING PROCEDURE. TETRA-COMPARTMENTAL MODEL

When the drugs are administered as eye drops the tears wash quickly the corneal surface, so that the drug is pulled to the nasal conduit and its corneal permeation also diminishes quickly.

This behaviour was reproduced in our experiments subjecting the initial endothelial drug solution to a continuous wash of artificial tear during the permeation measures. The graphic so obtained is shown in Fig. (**4**).

The apparent permeability calculated in this graphic by the slope at sixty minutes in both procedures have the values shown in the figure.

This model requires a more complex treatment as a tetracompartmental pharmacokinetic model.

In this model the statement of corneal permeability requires three differential equations:

$\frac{dC_{c}}{\mathrm{dt}}=k_{1}\left(C_{e}-C_{c}\right)-k_{2}\left(C_{c}-C_{i}\right)$

$\frac{dC_{i}}{\mathrm{dt}}=k_{2}\left(C_{c}-C_{i}\right)$

$\frac{dC_{e}}{\mathrm{dt}}=-k_{1}\left(C_{e}-C_{c}\right)-k_{3}\cdot C_{e}$

The main variation of Ce is to wash by ATS so it can approach $\frac{dC_{e}}{\mathrm{dt}}\text{to}\frac{dC_{e}}{\mathrm{dt}}=-k_{3}\cdot C_{e}$

So the anterior equation of the tricompartmental model is reduced to the following:

$C_{i}+\left(k_{1}+2\cdot k_{2}\right)\cdot C_{i}+k_{1}\cdot k_{2}\cdot C_{i}=k_{1}\cdot k_{2}{\cdot C}_{e}$

And with a similar treatment that tricompartmental model let us to:

$C_{i}\left(t\right)=A\cdot \left(e^{-n_{1}\cdot t}-e^{-n_{2}\cdot t}\right)+B\cdot \left(e^{-k_{3}\cdot t}-e^{-n_{2}\cdot t}\right)$

Were “t” is also the variable time and n_1_, n_2,_ A and B, pharmacokinetic parameters related to k_1_, k_2_ and k_3_.

## TRANSCORNEAL PERFUSION OF DICLOFENAC β-CYCLODEXTRIN COMPLEX

In ophthalmology, local drug administration in the form of topically applied low viscosity aqueous eye drop solutions is generally preferred.

Topically applied drugs must be, at least to some degree, soluble in the aqueous tear fluid. NSAIDs used to treat ocular inflammation are lipophilic water-insoluble compounds that have to be introduced into aqueous eye drop formulations as water-soluble salts (like sodium diclofenac salt). However, they must also be somewhat lipid-soluble in order to penetrate the lipophilic corneal epithelium, through the corneal stroma and the lipophilic endothelium into the aqueous humor. In both cases, ocular bioavailability is seriously hampered by the low aqueous solubility or the hydrophilic properties of the penetrating molecules, respectively. In other words, for successful formulation in an aqueous eye drop solution a drug must be both water-soluble (that is hydrophilic) and lipid-soluble (that is hydrophobic).

Cyclodextríns are novel, chemically stable adjuvants that enhance ocular bioavailability of ophthalmic drugs without affecting the barrier function of the eye or increasing the viscosity of the aqueous, eye drop formulation.

Cyclodextrins are a group of structurally related natural products formed during bacterial digestion of cellulose. These cyclic olígosaccharides consist on linked glucopyranose units with a hydrophilic outer surface and a lipophilic central cavity. The natural α-β and γ-cyclodextrins consist on six, seven and eight glucopyranose units, respectively. The internal cavity allows to lodge drug molecules with adequate dimensions [10].

In Fig. (**6**) the tridimensional structure of the diclofenac/ β cyclodextrin complex obtained by molecular modelling can be seen in our laboratory.

The cyclodextrin complexation of a drug molecule changes the physico-chemical properties of the drug, such as its aqueous solubility and chemical stability. Since the cyclodextrin molecule is hydrophilic on the outside, the complex formation usually increases the water-solubility of lipophilic water-insoluble drugs.

Thus, it is possible through cyclodextrin complexation to formulate lipophilic water-insoluble molecules as aqueous eye drop solutions. Furthermore, the chemical stability of the drug molecule is enhanced by the formation of the inclusion complex.

If the complex is located in close approximation to a lipophilic biological membrane (such as the eye cornea), the guest may be transferred to the matrix with the highest affinity. Importantly, since no covalent bonds are formed or broken during the guest-host complex formation, the complexes are in dynamic equilibrium with free drug and cyclodextrin molecules [11]. So, the hydrophilic cyclodextrins act as true carriers by keeping the lipophilic water-insoluble drug molecules in solution and delivering them to the membrane surface where the drug moves from the cyclodextrin cavity into the lipophilic membrane due to the membrane affinity to lipophilic drugs. The relatively lipophilic membrane has low affinity to large hydrophilic cyclodextrin molecules or hydrophilic drug/cyclodextrin complexes which remain in the aqueous exterior tear fluid [12].

To obtain the diclofenac/β-cyclodextrin complex 376 mg of sodium diclofenac and 1135 mg of β-cyclodextrin were mixed in a mortar. Then a few drops of water were added kneading during 15-30 min. to obtain a uniform paste. The resulting dough was poured on a metallic surface and dried in a furnace at 40º C during 24 hours. The dried product was crushed in a mortar and sieved to obtain a fine powder. The concentration of the diclofenac in the complex was determined by UV spectroscopy. Solutions of the diclofenac/β-cyclodextrin complex so prepared containing 0.1% of diclofenac in ATS were obtained to measure the drug corneal permeation.

The graphic of Fig. (**7**) reflects the corneal permeations of sodium diclofenac 0.1 percent solution in artificial tear with tears washing and the solution in this dissolvent of the diclofenac/ β-cyclodextrin complex at the same diclofenac concentration and identical procedure. It shows an important increase of corneal permeation with the latter.

The apparent permeability coefficients calculated in this graphic by the slope at sixty minutes in both formulations are 4.13·10^-4^ cm/h for the sodium diclofenac solution and 2.57·10^-3^ cm/h. for the diclofenac / β-cyclodextrin complex. This reflects that the amount of diclofenac transferred through the cornea is six times higher when the drug is forming a complex with β-cyclodextrin.

## CORNEAL PERMEATION OF FLURBIPROFEN NANOPARTICLES

Nanoparticles are polymeric particles smaller than one micrometer that can load drug to drive it to the site of action (organ or tissue) improving its bioavailability.

There are two forms of nanoparticles: nanospheres and nanocapsules. Nanospheres have a rigid structure forming a polymeric network or matrix. The drug is adsorbed on the surface, dissolved or included in the matrix. Nanocapsules have vesicular structure formed by a polymeric cover and a lipid nucleus (or core). The drug is generally solved or included in the nucleus.

Polymeric nanoparticles can improve the specificity of action, cellular penetration, and protection against the inactivation of drugs. The main interest of these nanosystems is the possibility to modify the pharmacokinetic behaviour of drugs.

In this work the corneal permeation of flurbiprofen, a slightly water-soluble drug, loaded in nanospheres of two different polymers: polylactic-polyglycolic acid and poly-ε-caprolacton were assayed.

Flurbiprofen (FB), loaded nanospheres of the above named polymers were prepared. They were made by the solvent displacement technique described by Fessi and collaborators [13].

Brieﬂy, an organic solution of 90 mg of polymer in 25 mL of acetone containing FB (from 0.16 to 1.84 mg/mL) was poured, under moderate stirring into 50 mL of an aqueous solution adjusted to the desired pH value (2.82– 6.18) from 6.60 to 23.40 mg/mL of poloxamer 188 (P188). Acetone was then evaporated and the volume of NPs dispersion was concentrated to 10 mL under reduce pressure (Bu¨ chi B-480, Flawil, Switzerland). Empty NPs with or without P188 were prepared according to the same procedure but omitting the addition of FB in the organic phase. Table **1** describes the physicochemical parameters of Flurbiprofen loaded to polylactic-glycolic acid and poly-ε-caprolacton- nanospheres prepared at the same concentration as the commercial eye drops to perform the *ex vivo* permeation studies.

All formulations showed an average particle size near to 200 nanometers which is appropriate for ophthalmic application. The particle size distribution was very narrow in all cases as the polidispersity index was less than 0.1 (zero point one), corresponding to monodisperse systems. The entrapment efficiency of these nanospheres was found to be around eighty five per cent for Flurbiprofen – polylactic-glycolic acid and around ninety seven per cent for Flurbiprofen loaded poly-ε-caprolacton [14].

Flurbiprofen -loaded polylactic-glycolic acid or poly-ε-caprolacton nanospheres containing Flurbiprofen were tested and compared with the free drug solution in phosphate buffer solution, pH 7.4.

The transcorneal permeation profile of flurbiprofen is shown in Fig. (**8**).

The permeation coefficient values calculated for each formulation confirmed these results. Flurbiprofen -loaded polymer nanoparticles showed a significantly higher drug permeation capability compared with the drug.

## CONCLUSIONS

A useful procedure to determine the corneal drug permeation has been developed.

The formation of NSAIDs with cyclodextrins has been as an a effective procedure to increase remarkably the bioavailability of the anti-inflammatory drugs.

The efficiency of polymeric nanoparticles of polylactic-polyglycolic acid and the poly-ε-caprolacton as intraocular targeting of NSAIDs has also been proved, being the latter polymer more effective to increase the corneal permeation of the assayed drug flurbiprofen.

## Figures and Tables

**Fig. (1):**
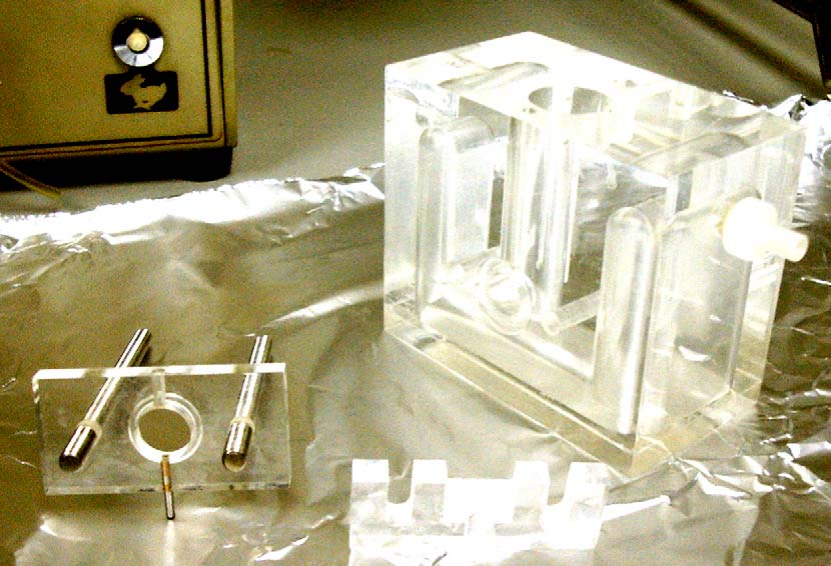
Detail of the metacrylate chamber to clamp the cornea.

**Fig. (2):**
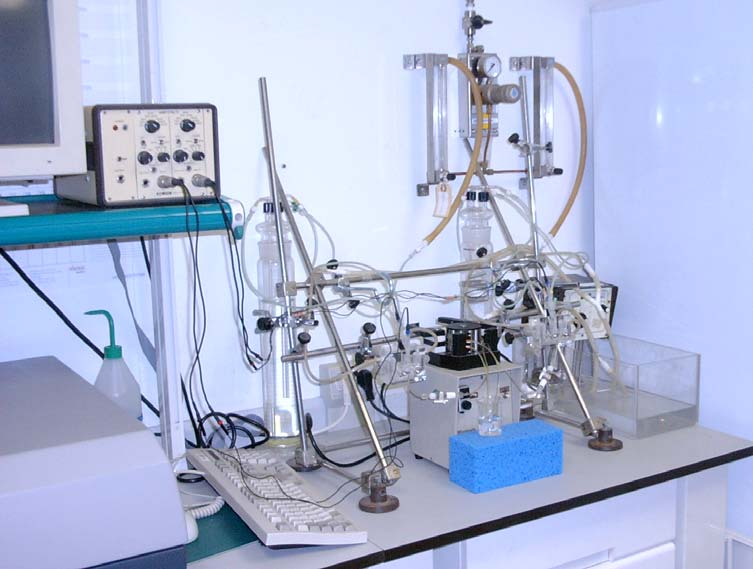
Entire setup of the corneal device for the study of corneal permeation.

**Fig. (5):**
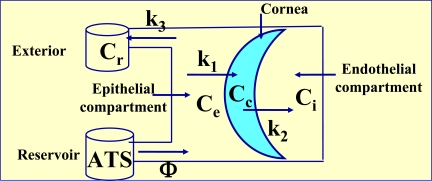
Tetracompartmental model. Ce is now the variable external concentration, Cr is the residual also variable concentration and K3 the transference constant from epithelial compartment to exterior. Cc, Ci, K_1_ and K_2_ have the same significance as the anterior model.

**Fig. (6):**
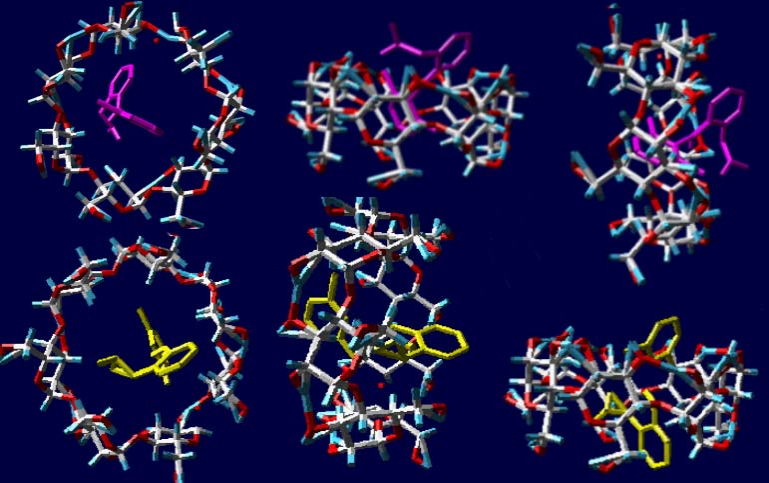
Molecular modelling obtained in our laboratory of the diclofenac β–cyclodextrin complex [1].

**Fig. (7):**
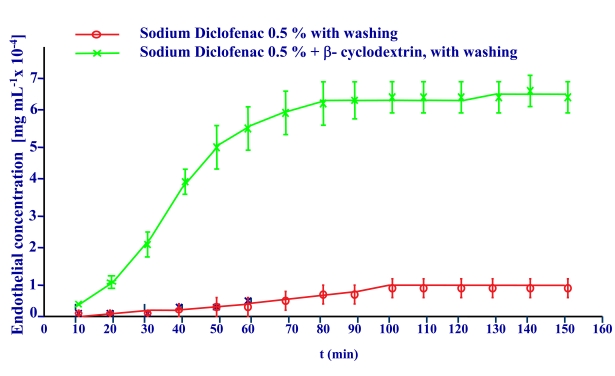
Cornea permeation profile of Sodium Diclofenac 0.5% free drug solution and of the Diclofenac/β-cyclodextrin complex at the same diclofenac concentration with washing procedure.

**Fig. (8):**
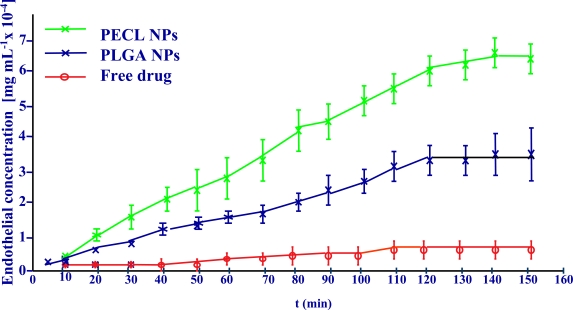
Corneal permeation of flurbiprofen loaded nanospheres.

**Table 1:** Composition and Physicochemical Properties of Flurbiprofen Loaded Nanospheres of Polylactic-Glycolic Acid (FB-PLGA) and Poly-ε-Caprolacton (FB-PCL)

Nanospheres	Zav ± SD (nm)	PI ± SD	EE ± SD (%)
FB-PLGA	209.9 ± 6.4	0.039 ± 0.027	85.94 ± 1.3
FB-PCL	200.1 ± 7.1	0.060 ±0.019	97.16 ±2.1

Z_av_: Average particle size. PI: Particle size distribution. EE: Entrapment efficiency. SD: Standard deviation.

**Table 2:** Apparent Permeation Values of Flurbiprofen Free Drug and Loaded Nanospheres

Flurbiprofen	Papp (cm/s x 10^6^)
**Free drug**	2.48 ± 1.06
**PLGA NPs**	18.98 ± 0.65
**PECL NPs**	9.78 ± 12.19

Flurbiprofen - poly-ε-caprolacton nanospheres reveal a major permeation coefficient than polylactic-glycolic acid nanospheres.

## References

[1] Valls R (2008). Thesis, University of Barcelona.

[2] Camber O (1985). Acta Pharm. Suec.

[3] Diepold R, Kreuter J, Himber J, Gurny R, Leen VHL, Robinson JR (1989). Graefe's Arch. Clin. Exp. Ophthalmol.

[4] Myung D, Derr K, Huie P, Noolandi J, Ta K, Ta C (2006). Ophthalmic Res.

[5] Donn A, Maurice DM, Mills NL (1959). Arch. Ophthalmol.

[6] Ehlers N, Ehlers D (1966). Acta Ophthalmol.

[7] O'Brien WJ, Edelhauser HF (1977). Invest. Ophthalmol. Vis. Sci.

[8] Grass GM, Robinson JR (1988). J. Pharm. Sci.

[9] Maurice DM, Mishima S, Sears ML (1984). Ocul Pharmacokinet. “Pharmacology of the eye”.

[10] Szejtly J (1984). Cyclodextrin Tecnol. Topics in Inclusion Science.

[11] Loftsson T, Järvinen T (1999). Adv. Drug Deliv. Rev.

[12] Loftsson T, Stefánsson E (2002). Acta Opthalmol. Scand.

[13] Fessi H, Pusieux F, Devissaguet JP, Ammoury M, Benita S (1969). Int. J. Pharm.

[14] Alonso MJ, García ML, Espina M, Valls O, Egea MA (2000). Boll. Chim. Farmac.

